# Characterization of sensory neuronal subtypes innervating mouse tongue

**DOI:** 10.1371/journal.pone.0207069

**Published:** 2018-11-08

**Authors:** Ping Wu, Dominic Arris, Max Grayson, Chia-Nung Hung, Shivani Ruparel

**Affiliations:** 1 Department of Endodontics, University of Texas Health at San Antonio, San Antonio, TX, United States of America; 2 Department of Molecular Medicine, University of Texas Health at San Antonio, San Antonio, TX, United States of America; 3 Department of Life Science, Tunghai University, Taichung, Taiwan; National Eye Centre, UNITED STATES

## Abstract

The tongue is uniquely exposed to water-soluble environmental chemicals that may lead to injury or tumorigenesis. However, comparatively little research has focused on the molecular and functional organization of trigeminal ganglia (TG) afferent neurons innervating the tongue. The current study identified and characterized lingual sensory neurons based on a neuronal subtype classification previously characterized in the dorsal root ganglion (DRG) neurons. We employed immunohistochemistry on transgenic reporter mouse lines as well as single-cell PCR of known markers of neuronal subtypes to characterize neuronal subtypes innervating the tongue. Markers expressed in retrogradely labeled TG neurons were evaluated for the proportion of neurons expressing each marker, intensity of expression, and overlapping genes. We found that tongue-innervating sensory neurons primarily expressed CGRP, TRPV1, TrkC, 5HT3A and Parvalbumin. These markers correspond to peptidergic and a subgroup of non-peptidergic C-nociceptors, peptidergic A nociceptors, proprioceptors and myelinated low-threshold mechanoreceptors (LTMRs). Interestingly, as reported previously, we also found several differences between TG and DRG neurons indicating the need for single-cell sequencing of neuronal types based on tissue type within all TG as well as DRG neurons.

## Introduction

Several abnormalities of the tongue cause pain and impact quality of life. Many lingual injuries are transient such as in oral cankers, lacerations and cold sores, while others like oral thrush and glossitis can last a few weeks [1–6]. On the other hand, conditions like tongue neuralgia, burning mouth syndrome, oral cancer and mucositis can last from months to even years leading to a significant reduction in quality of life of these patients [7–16]. Therefore, knowledge of lingual nociceptive mechanisms is critical to be able to treat these patients safely and effectively. The majority of lingual sensory innervation is conveyed via TG afferents in the lingual nerve terminating in the anterior two-thirds of the tissue, whereas the posterior one-third is innervated by the glossopharyngeal nerve [17–19]. The lingual nerve arises from the mandibular division of the trigeminal nerve and its cell bodies lie in the V3 branch of the trigeminal ganglion (TG). The traditional histologic classification of afferent neurons consists of three primary groups: the unmyelinated C fibers, the lightly myelinated Aβ fibers and the heavily myelinated Aδ fibers. However, molecular and functional studies on afferent neurons of the dorsal root ganglia (DRG), have shown that sensory fiber types can be divided into subgroups based on their gene expression profiles or specific functions and that these subgroups can be identified by the expression of certain specific markers [20–24]. Expression and regulation of these sensory neuronal subgroups can therefore be studied individually, providing detailed insight into the physiological actions of these neurons in normal and diseased states. However, no study has identified the different types of sensory neurons innervating the tongue. Using retrograde labeling, transgenic reporter mice, immunohistochemistry and single-cell PCR, this study identifies the types of sensory neuronal subgroups in tongue innervated by TG neurons and provides the first report of the molecular classification of lingual afferents.

## Materials and methods

### Animals

The animal protocol was approved by the UTHSCSA IACUC and conforms to IASP guidelines. 8-12-week-old adult male and female transgenic animals were used for all experiments. Table 1 lists all the transgenic phenotypes used for the study. Rosa26-LSL-TdTomato, parvalbumin (PV-Cre) and TrkB-CreERT2 mouse lines on B6.129 background, were obtained from the Jackson Laboratory (Bar Harbor, ME). 5HT3A-GFP transgenic mouse line was purchased from the GENSAT program (MMRRC services; UNC, NC and UC Davis, CA, respectively). MRGPRA3-Cre and transgenic NPY2R-tdTomato mouse lines were kindly provided by Dr. Xinzhong Dong (John Hopkins University Medical School, Baltimore, MD). MRGPRD-GFP knock-in mouse line was kindly provided by Dr. Qin Liu (Washington University, St. Louis, MO). TrkC-CreERT2 mouse line was generated in Dr. David Ginty’s laboratory (Harvard Medical School, Boston, MA) and kindly provided by Dr. Yu Shin Kim (UTMB, Galveston, TX). TrkB and TrkC reporter mouse lines were inducible lines for which recombination was induced by injecting 80mg/kg tamoxifen (Sigma-Aldrich, St. Louis, MO) intraperitoneally, four times, every other day. Recombination and induction were allowed to occur for at least 10 days following last tamoxifen injection before the animals were used for experiments.

**Table 1:** List of transgenic mouse lines used in the study.

*Phenotype*	*Provided by*	*Generated by*	*References*
MRGPRD-GFP	Dr. in Liu		[25, 26]
MRGPRA3-GFP-TDT	Dr. Xinzhong Dong	Crossed MRGPRA3-GFP with Rosa 26^LSL-tdtomato/+^	[27]
5HT3A-GFP	GENSAT Program		[28]
TrkB-ER-TDT		Crossed TrkB^CreERT2^ (Jackson Laboratory) with Rosa 26^LSL-tdtomato/+^	[24]
NPY2R-TdT	Dr. Xinzhong Dong		[29]
TrkC-ER-TDT	Dr. Ru Shin Kim	Crossed TrkC^CreERT2^ (Jackson Laboratory) with Rosa 26^LSL-tdtomato/+^	[30]
Parv-TDT		Crossed PV^Cre^ Jackson Laboratory) with Rosa 26^LSL-tdtomato/+^	[31]

### Retrograde labeling

Mice were briefly anesthetized by isoflurane inhalation and lingual neurons were labeled by bilateral injections of 5ul of 1% WGA-488 (ThermoFisher Scientific, Carlsbad, CA) in td-tomato (TDT) expressing mice and 1% WGA-568 in GFP expressing mice. WGA was injected twice on each side of the tongue; once superficially and once deeper in the tissue, to maximize labeling of lingual neurons. The two injections were spaced at a 4h interval. Mice were used for experiments three days later.

### Immunohistochemistry

Mice were deeply anesthetized with an i.m. injection of ketamine (75mg/kg) and dexmedotomidine (1mg/kg), and transcardially perfused with 30ml of 4% paraformaldehyde (PFA) in 0.1M phosphate buffer (PB). The TG were removed, post-fixed for 20 min, rinsed in PB and placed in cold 0.1M PB with 10% sucrose for 6 hours and then 30% sucrose overnight. Tissues were embedded in Neg-50 (Richard Allan, Kalamazoo, MI, USA) and sectioned in the horizontal plane at 20μm with the use of a cryostat. Sections were placed onto Superfrost Plus slides (Fisher Scientific, Waltham, MA, USA), dried and stored at −20°C until stained. Immunostaining was performed as described previously [13, 32, 33]. Briefly, tissue sections were permeabilized and blocked for non-specific protein binding with blocking solution consisting of 4% normal goat serum (Sigma, St. Louis, MO), 2% bovine gamma- globulin (Sigma-Aldrich, St. Louis, MO) and 0.3% Triton X-100 (Fisher Scientific) in PBS for 90min prior to incubation with primary antibodies in blocking solution for 16h. Table 2 lists the antibodies, dilutions and sources used in this study. Sections were rinsed with PBS, incubated in secondary antibody in blocking solution for 90min, rinsed in PBS and H2O, dried and coverslipped with Vectashield. Secondary antibodies were purchased from Molecular Probes, Eugene, OR, USA and were used at a dilution of 1:200 for all experiments. All immunostaining procedures were performed at room temperature. Sections were evaluated, and images obtained with a Nikon Eclipse 90i microscope equipped with a C1si laser scanning confocal imaging system. Multiple z-stack images were acquired of the V3 region of TGs using three different sections of two TGs per animal from 2–3 individual animals for each antibody combination with a 20x objective and identical laser gain settings. Images were taken using fixed acquisition parameters across all groups and were unaltered from that initially taken. Quantitation was achieved using NIS Elements software for number of neurons above threshold, in the V3 region of the TG tissue in each image. A total of 150–300 neurons per animal were counted.

**Table 2:** List of antibodies used in the study.

Primary Antibody	Dilution	Company	Secondary Antibody	References
CGRP	1:300	Sigma -Aldrich	Goat-Anti Rabbit AlexaFlour 405	[34–36]
TRPV1	1:400	Neuromics, Edina, MN	Goat-Anti Guinea Pig AlexaFlour 405	[37–41]
Calbindin	1:500	Swant Antibodies, Switzerland	Goat-Anti Rabbit AlexaFlour 405	[20, 42]
TH	1:400	Pel-Freez, Rogers, AR	Goat-Anti-Rabbit AlexaFlour 568	[43, 44]

CGRP: calcitonin-gene related peptide; TRPV1: transient receptor potential vanilloid 1; TH: tyrosine hydroxylase

### Primary cultures of TG neurons

Mice were deeply anesthetized with an i.m. injection of ketamine (75mg/kg) and dexmedetomidine (1mg/kg) and transcardially perfused with 30ml of ice cold 0.1M PB. The V3 region of TG tissue were dissected and kept in ice cold Hank's Balanced Salt Solution (HBSS). The TGs were transferred to HBSS containing 50 ng/mL collagenase (type 1, Sigma) and 50 ng/mL dispase (type 2, Sigma) and incubated for 45min at 37°C. After enzymatic digestion, the samples were centrifuged for 2min at 1,000 rpm and washed using Dulbecco's modified eagle medium with 10% fetal calf serum and 1% penicillin/streptomycin. After the samples were centrifuged for 2min at 1,000 rpm again, the pellet was re-suspended in growth media. Cell suspension was filtered through a 70 μm cell sieve (Falcon) to remove pieces of tissue that were not dissociated and plated on a 6-well plate on ice and transferred to Bioanalytic and Single-cell core (BASiC) at UT Health San Antonio immediately for single-cell isolation.

### Single-cell PCR

Experiments were performed based on protocols described previously [45] with some modifications. Briefly, an aliquot of cell suspension was transferred into an uncoated sterile petri dish and WGA-488 positive single neuron cells were isolated individually using a Narishige micromanipulator and FERTY Syringe Plus Microinjector under an inverted EVOS FL digital fluorescence microscope (AMG, Bothell, WA). Each cell was placed into a 0.2 ml PCR tube containing 4μl of 2X reaction mix from CellsDirect^TM^ One-Step qRT-PCR Kit (Thermofisher Scientific) and immediately frozen on dry ice and then stored at -80C until further use. Cells were also measured in size and classified as small for <15 microns, medium for <25 microns and large for >25 microns in size. Target genes were amplified with Fluidigm PreAmp master mix and TaqMan probes (Applied Biosystems, Table 3). A 200pg sample of Universal Mouse RNA (BioChain, Newark, CA) and no-template control (NTC) were used as positive and negative controls. In addition, PreAmp NTC, involving amplification of only the primer mixture was used as the quality control for all primers. Gene Expression analyses (qRT-PCR) were performed with the 48.48 IFCs (integrated fluidic circuits) using BioMark^TM^ HD system (Fluidigm, Inc.) according to manufacturer’s protocol. The cycles of threshold (Ct) values were collected using GE 48x48 Standard v.pcl program (Fluidigm, Inc.). Ct value per gene per cell was normalized to its internal control gene, UBB. Relative gene expression levels were obtained using 2ˆ (-delta Ct) formula.

**Table 3:** List of genes and primers for single-cell PCR.

Gene	Catalog Number
TRPV1	Mm0124601_m1
CGRP	Mm00801462_m1
5HT3A	Mm00442874_m1
NFH	Mm01191455_m1
Calbindin (CALB1)	Mm00486647_m1
NPY2R	Mm01218209_m1
Parvalbumin (PVALB)	Mm00443100_m1
MRGPRD	Mm04212994_m1
MRGPRA3	Mm01612005_m1
vGLUT3	Mm00805413_m1
TH	Mm01268875_m1
TrkC	Mm00456222_m1
TrkB	Mm00435422_m1
CHRNA3	Mm00520145_m1
TRPA1	Mm01227437_m1
UBB	Mm01622233_g1

### Statistics

For immunohistochemical studies, data are presented as mean ± standard error of mean (SEM) for percentage of retrogradely labeled neurons expressing each of the tested markers. For single-cell PCR experiments, data are presented in the form of a heatmap for log_2_ values of 2^ (delta Ct). In addition, the intensity of expression for each gene between small, medium and large neurons is presented as median ± 95% confidence intervals of the relative mRNA expression. Statistical analysis was performed using parametric one-way ANOVA or non-parametric Kruskall-Wallis Test with Bonferroni correction in GraphPad Prism. P<0.05 was considered significant.

## Results

**Fig 1:**
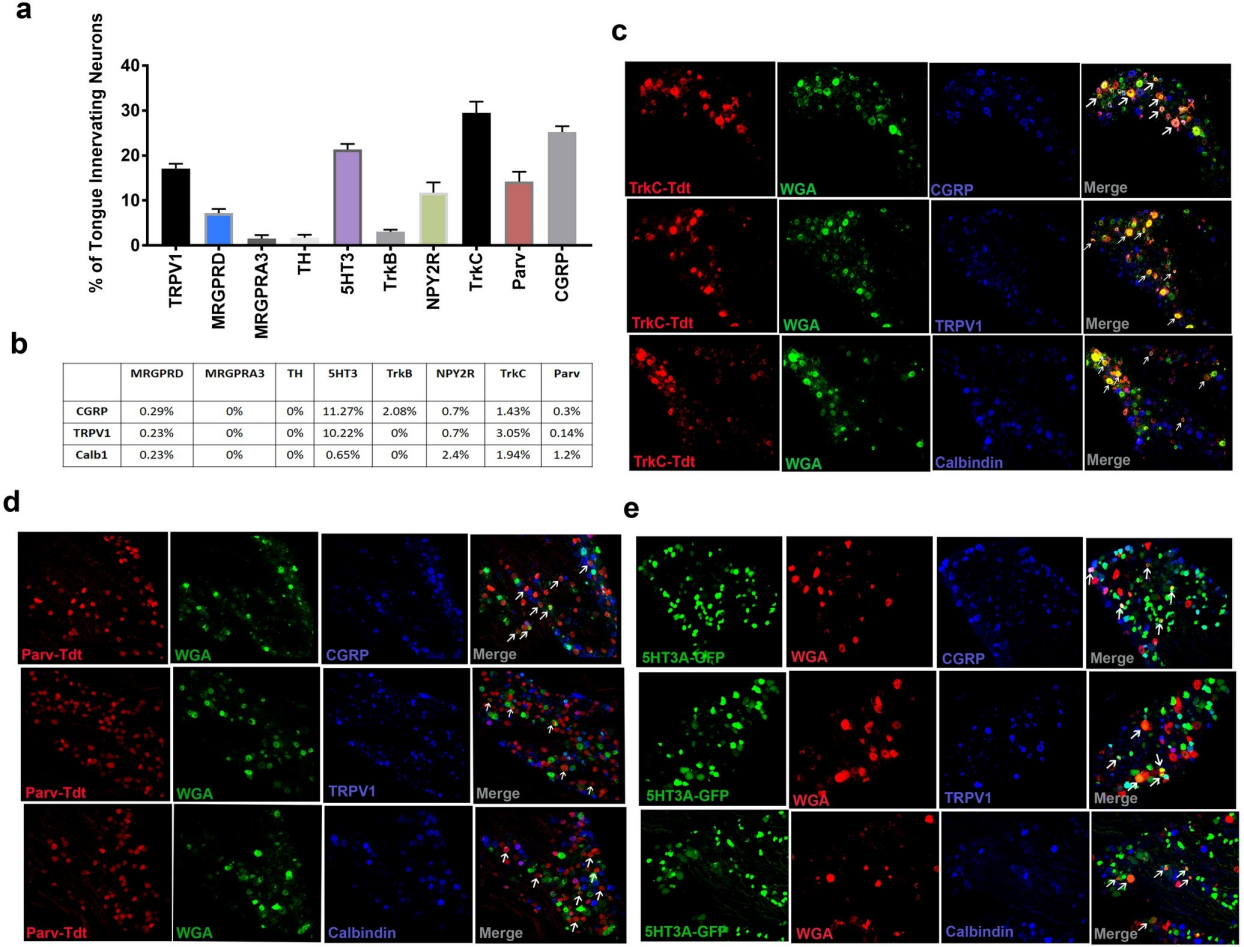
Expression of subtype markers in lingual sensory neurons. 1% WGA-488 or WGA-568 was injected in tongues of mice bilaterally and 3 days later, TG tissues were harvested for immunostaining. Transgenic reporter mouse lines were used to determine the types of sensory neuron innervating mouse tongue. **a.** Number of Neurons expressing the marker and co-localizing with WGA were counted from 2–3 animals per group. Data are presented as mean +/- SEM of percentage of neurons expressing each marker. **b.** Each TG tissue sections from each retrogradely labeled transgenic mouse was stained with antibodies specific for either TRPV1, CGRP or Calbindin. Data are presented as WGA-labeled neurons expressing both markers. **c**. Representative immunostaining of TrkC-TDT, WGA and TRPV1, CGRP or Calbindin. **d.** Representative immunostaining of Parv-TDT, WGA and TRPV1, CGRP or Calbindin.E. Representative immunostaining of 5HT3A-GFP, WGA and TRPV1, CGRP or Calbindin. Arrows indicate colocalization of WGA and reporter gene.

To identify subtypes of lingual neurons, we first performed immunohistochemistry using transgenic animals expressing reporter genes under the promoter of the different markers for sensory neuron subtypes. Markers and the corresponding subtypes are listed in Table 4. Due to unavailability of TH- reporter mouse, we used antibodies to stain for the marker. The lingual neurons were retrogradely labeled by injecting 1% WGA in the tongue and three days later; TG tissue was harvested for immunohistochemistry. Retrogradely labeled TG sections for each transgenic group were stained with either anti-CGRP, Anti-TRPV1 or anti-calbindin antibodies to further characterize the subtypes into peptidergic or non-peptidergic, TRPV1 co-localization or identify rapidly adapting mechano-sensitive neurons [20, 46] respectively. Our data showed that ~25% percent of all retrogradely labeled neurons were peptidergic as reflected by CGRP expression and 17% were TRPV1^+^ neurons (Fig 1A). Among other subtype markers, major subclasses included 5HT3A^+^ at 21%, TrkC^+^ at 31% and parvalbumin^+^ at 14%. NPY2R expressing neurons were also observed at 12%. While, MRGRPD expressing neurons innervated the tongue at 7% of all lingual sensory neurons, other markers including MRGPRA3, TH, TrkB and calbindin were expressed in low numbers ranging from 1–3.5% of all retrogradely labeled lingual TG neurons (Fig 1A). Of the different subtype markers tested, 5HT3A-expressing neurons showed co-localization with CGRP and TRPV1 at 11.3% and 10.2% respectively (Fig 1B and 1E). A small percentage of all TrkB expressing neurons also showed co-localization with CGRP, but none with TRPV1 or calbindin. Similarly, 3% of all TrkC positive neurons co-localized with TRPV1, 1.4% with CGRP and 1.9% with calbindin (Fig 1B and 1C), whereas parvalbumin positive neurons only co-localized with calbindin at 1.2% (Fig 1B and 1D). Collectively, data from our anatomical analysis showed that sensory neurons that innervate mouse tongue are comprised of peptidergic and non-peptidergic neurons and of these, 5HT3A, TrkC, parvalbumin and TRPV1 positive neurons make up the majority of subtypes innervating the tongue.

**Table 4:** List of markers and the corresponding sensory neuronal subtypes.

Marker	Subtype
TRPV1	C-nociceptor
MRGPRD	C- nociceptor
MRGPRA3	C-nociceptor
CGRP	Peptidergic Neurons
TH	C-LTMR
5HT3	Peptidergic Aδ neurons
TrkB	Aδ-LTMR
NPY2R	A- nociceptors
TrkC	Aβ-LTMR
Parvalbumin	Proprioceptors

While use of immunohistochemistry of transgenic reporter animals provided information on the subtypes of sensory neurons in the tongue, it did not allow for detailed characterization of the different subgroups such as cell size distribution, myelination status of the subtypes, whether or not the markers overlap in expression with each other. Moreover, IHC analysis does not determine whether similar subgroups exist in the trigeminal system as compared with the DRG. To address these gaps, we performed single-cell PCR of retrogradely labeled lingual neurons that provided an independent validation of our data obtained from immunohistochemistry. We also tested for expression of additional genes that were not evaluated with immunohistochemistry, including CHRNA3; a marker for mechanically insensitive (MI) neurons [22], vGLUT3; a marker for C-LTMRs [47] and TRPA1 to further breakdown C-nociceptor subgroups based on a classification reported in mouse DRG [21, 23]. A solution of 1% WGA was injected into the tongues of wild-type C57BL6 mice and three days later, TG tissues were dissected and dissociated for manual picking of WGA-positive neurons. Neurons were also categorized as small, medium and large. Each cell was evaluated for the following genes using quantitative RTPCR: CALB1, CGRP, CHRNA3, 5HT3A, MRGPRA3, MRGRPD, NFH, NPY2R, TrkB, TrkC, Parvalbumin, vGLUT3, TRPA1, TRPV1 and TH.

**Fig 2:**
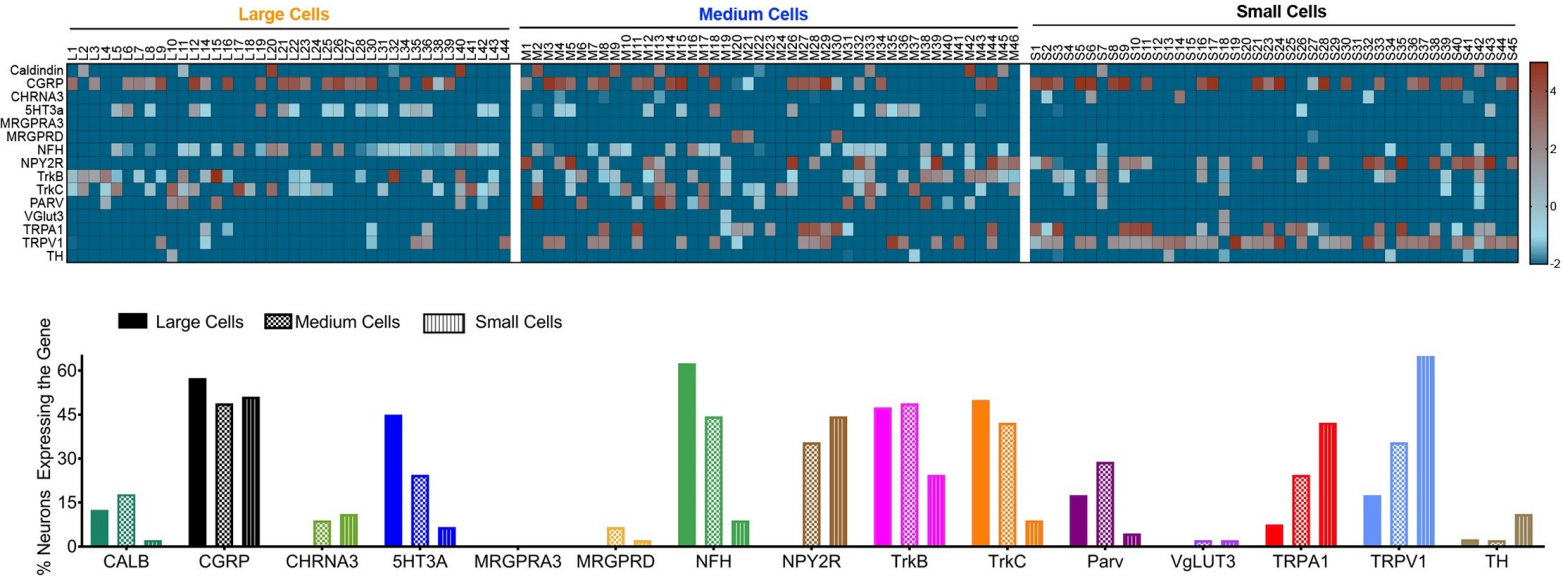
Expression profile of lingual neurons with single-cell PCR. Retrogradely labeled tongue-innervating TG neurons were manually picked to perform single-cell PCR of target genes. Gene expression was calculated for relative mRNA expression for each cell for each gene. **a.** Data are presented as heatmap for log2 values of the relative mRNA expression for each gene for each cell. UBB was used as an internal control. **b.** Number of neurons expressing each gene in large, medium and small cells were counted and presented as percentage of neurons expressing the target gene within each size group.

**Fig 3:**
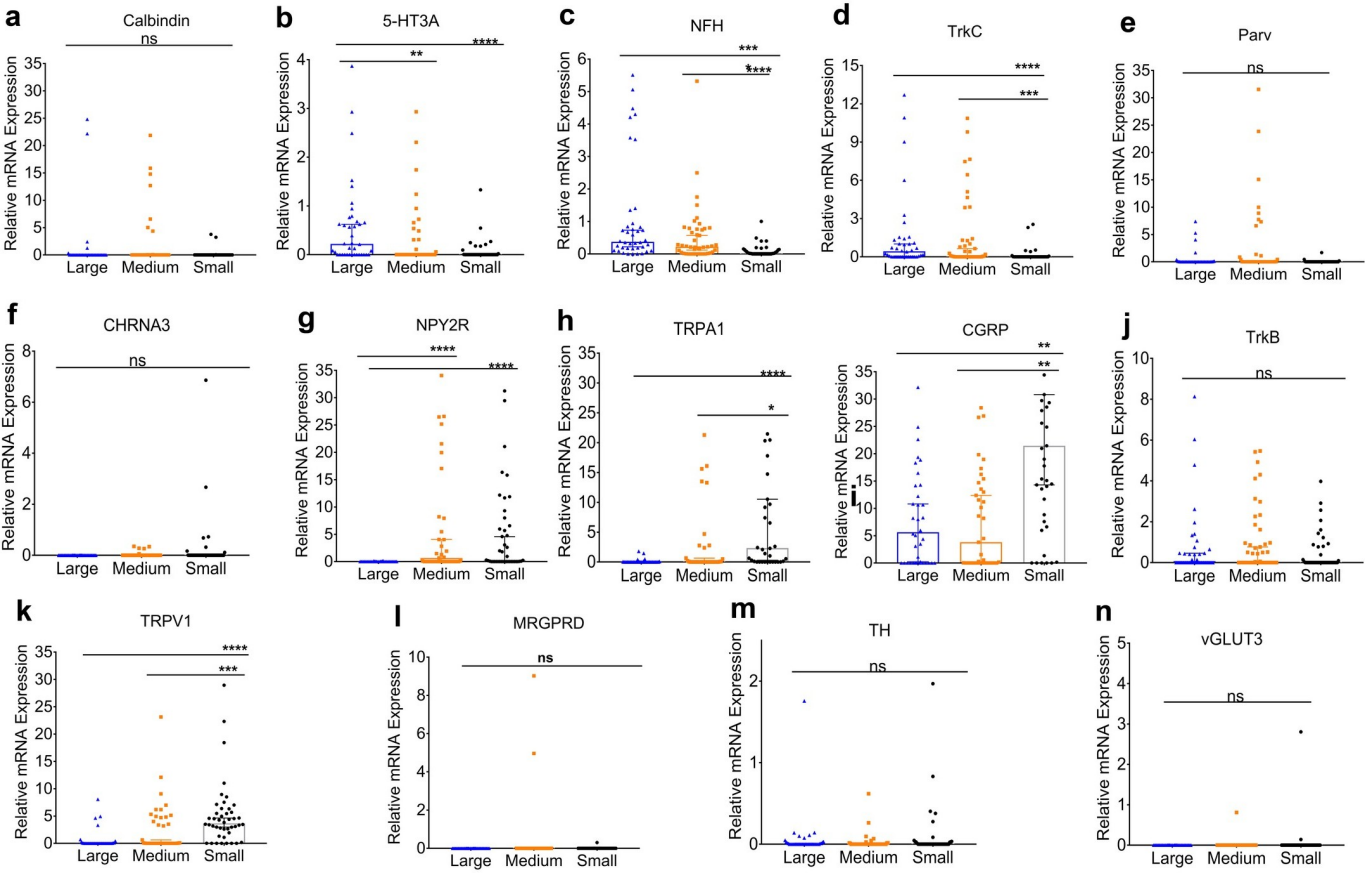
Intensity of expression of target genes based on sensory neuronal cell size. Relative mRNA expression values from single-cell PCR for each gene are presented as median +/- 95% confidence interval in large, medium and small neurons. Expression intensities for each gene was compared using parametric one-way ANOVA with Bonferroni post-hoc test, if the variances in means were not significant. If significant, non-parametric Kruskal-Wallis with Bonferroni post hoc test was used. p<0.05 was considered significant.

Calbindin was primarily expressed in 12.5% of large and 18% of medium sized neurons (Fig 2A and 2B) at similar levels of expression (Fig 3A). Similarly, 5HT3A was expressed in 45% large and 24.4% medium sized neurons (Fig 2A and 2B), with a significantly greater level of expression in large neurons compared to medium neurons (Fig 3B). The frequency of NFH positivity (Fig 2A and 2B) as well as the magnitude of its expression (Fig 3C) was seen significantly greater in large and medium sized compared to small neurons. While, Parvalbumin as well as TrkC expressed primarily in large and medium neurons (Fig 2A and 2B), only TrkC (Fig 3D), but not Parvalbumin (Fig 3E), showed greater levels of expression in large and medium neurons compared to small neurons. On the other hand, CHRNA3, NPY2R and TRPA1 were expressed primarily in small to medium sized neurons (Fig 2A and 2B). Of these, only TRPA1 showed significant differences in intensity of expression between medium and small neurons (Fig 3F–3H). CGRP was expressed in approximately equal numbers in large (57.5%), medium (49%) and small (51.5%) neurons (Fig 2A and 2B); however, the magnitude of its expression were significantly higher in small neurons compared to medium and large neurons (Fig 3I). Interestingly, unlike our observation with immunostaining of TG tissue from TrkB-TDT mice (Fig 1A), TrkB was expressed in small (25%), medium (49%) and large (47%) neurons (Fig 2A and 2B), with similar intensity in all neurons (Fig 3**J**). While the majority of TRPV1^+^ neurons was observed in small neurons, a substantial proportion was also seen in medium (35.5%) as well as large neurons (17.5%) (Fig 2A and 2B**)**. Levels of expression of TRPV1 were the greatest in small neurons, followed by medium sized neurons and the lowest levels were seen in the large neurons (Fig 3K). TH and MRGRPD, were only expressed in small neurons and medium to small neurons at low levels (Fig 2A and 2B). MRGPRA3 and vGLUT3 were expressed at 0–2% in lingual trigeminal neurons (Fig 2A and 2B).

**Fig 4:**
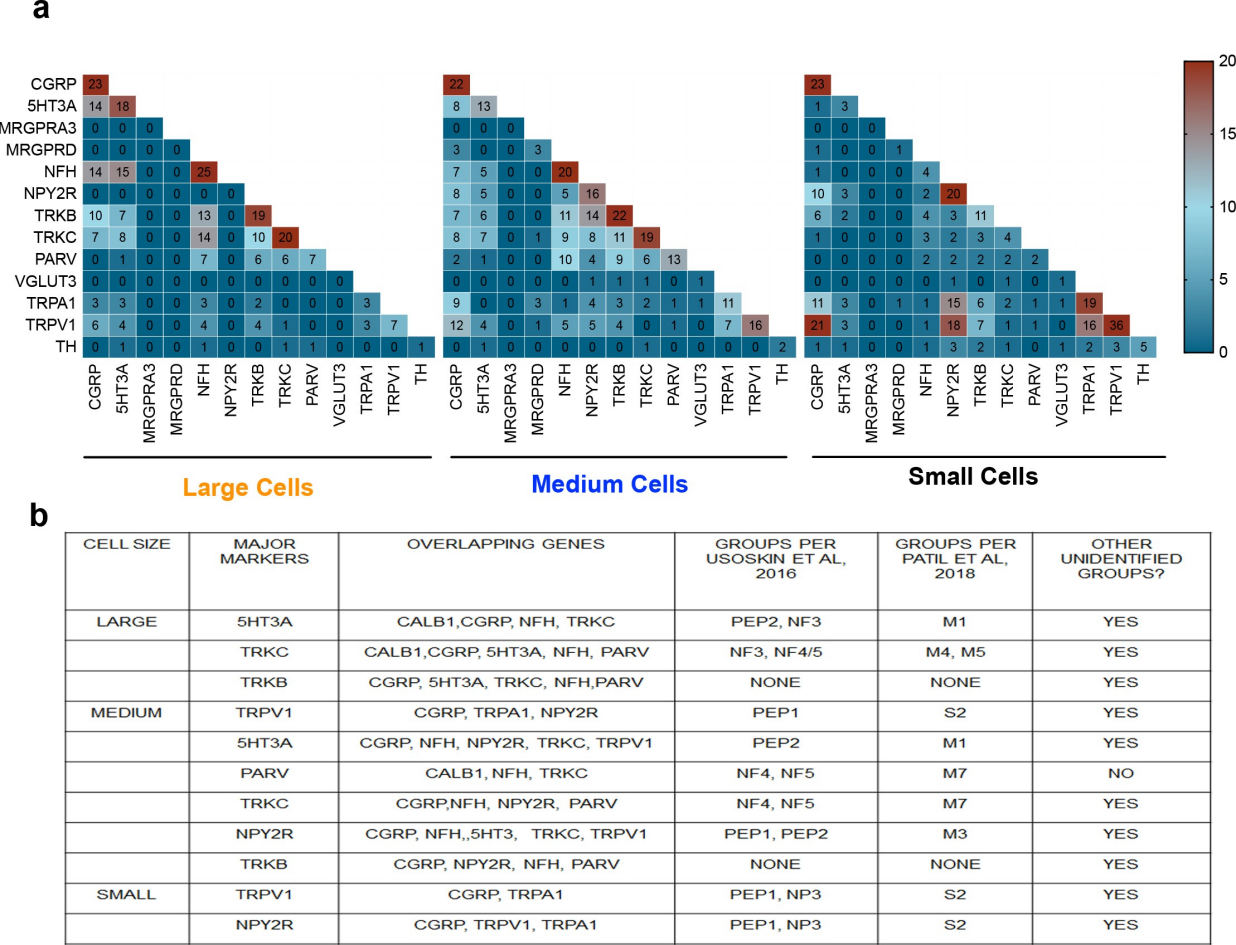
Identification of subgroups and overlapping markers in lingual sensory neurons. **a.** Heat map representing number of neurons expressing each gene in large, medium and small neurons. **b**. List of major markers expressed, overalapping genes in large medium and small neurons. Subgroups identified from Usoskin *et al* and Patil *et al* studies that correspond to the major markers is also listed.

We then analyzed the single-cell PCR data to determine overlapping genes among major markers expressed in large, medium and small neurons. As seen in Fig 4A and 4B, among the large neurons, major markers expressed were 5HT3A and TrkC. 5HT3 expressing large cells overlapped with Calbinidin, CGRP, NFH and TrkC expression. Similarly, TrkC expressing large cells co-expressed calbindin, CGRP, NFH, 5HT3A as well as parvalbumin. We also determined whether lingual neurons expressing the markers belonged to neuronal subgroups identified previously in the DRG neurons [21, 23]. As listed in Fig 4B, the majority of 5HT3A expressing large cells were identified with the PEP2 group from the Usoskin *et al* study and M1 group from Patil *et al* study. However, we also observed several 5HT3A expressing cells that did not belong to any identified groups such as peptidergic unmyelinated neurons lacking TRP channels. TrkC expressing large neurons were identified with NF3, NF4/5, M4 and M5 subgroups from DRG neurons (Fig 4A and 4B). Similar to 5HT3A, several TrkC expressing large neurons also were unidentified and included peptidergic unmylineated and mylineated neurons lacking TRPV1 and TRPA1 as well as non-peptidergic C neurons that did not fall under the TH group in the Usoskin *et al* study.

Among the medium sized lingual neurons, TRPV1, 5HT3A, parvalbumin, TrkC and NPY2R were primarily expressed. TRPV1 expressing medium neurons co-expressed with CGRP, TRPA1 and NPY2R (Figs 4A and 3B). Unlike the large neurons, 5HT3A medium sized neurons co-expressed CGRP, NFH, NPY2R, TrkC and TRPV1. Parvalbumin neurons overlapped with calbindin, NFH and TrKC whereas NPY2R expressing neurons overlapped with the majority of genes including CGRP, NFH, 5HT3A, TrkC and TRPV1 (Figs 4A and 3B). Few of the TRPV1 positive medium neurons belonged to the PEP group from the Usoskin study or S2 group from the Patil *et al* study. Other TRPV1 positive neurons did not belong to any previously identified groups; however, many cells showed a profile similar to the NP3 group from the Usoskin study such as TRPA1 and CGRP positivity and NFH negativity without MRGPRA3 expression. Like the large neurons, 5HT3A positive medium neurons belonged to PEP2 group, although in fewer numbers than the large neurons. Most other 5HT3A positive neurons were not classified in the identified groups. Majority of the TrkC positive medium neurons were identified to be in the NF3 or NF4/5 subgroups corresponding to the M7 group from Patil *et al* study. Interestingly TrkC positive neurons were also seen in some peptidergic and non-peptidergic C neurons. All Parvalbumin medium neurons fitted under the NF4/5 or M7 subgroups (Figs 4A and 3B). A few of NPY2R positive neurons corresponded to PEP1 and PEP2 or M3 subgroups; however, majority were unidentified. Notably, TrkB was expressed as a major marker in both large and medium neurons, although did not belong to any of the previously classified subgroups from the DRG neurons (Figs 4A and 3B).

Two markers; namely TRPV1 and NPY2R were expressed in small neurons innervating the tongue (Figs 4A and 3B). One-third of TRPV1 positive small neurons corresponded to the PEP1 or the S2 groups followed by the NP3 subgroup. Similar to medium TRPV1 neurons, NP3-like subgroup was also observed in small neurons and the remaining were unidentified. Interestingly, a third of NPY2R expressing small neurons also belonged to the NP-3 like subgroup while a few belonged to NP3 and PEP1 (or S2) and others were unidentified.

## Discussion

Recently, several studies have classified sensory neurons into various subgroups based on gene expression profiles and/or functional responses. Single-cell sequencing results from prior studies reveal that the major differences in genetic makeup lie in CGRP expression, TRPA1 and TRPV1 expression, myelination status as well as specific markers that further categorize the primary groups into a variety of subgroups. Usoskin *et al*, classified DRG sensory neurons into 11 different subtypes and identified important genes that are specifically expressed in each subgroup [23]. For example, TH and vGLUT3 are primarily expressed in C-LTMRs whereas MRGRPD and MRGPRA3 are found in two separate subgroups of the unmyelinated C nociceptors, parvalbumin in proprioceptors, TrkC in Aδ LTMRs and TrkB in Aβ LTMRs. Several of these markers have also been identified on a functional level by other groups [4, 24, 48]. Moreover, recent study by Patil *et al* functionally and anatomically corroborated these results and reported additional categorization of DRG sensory neuronal subtypes [21]. Other studies have identified additional markers on a functional basis for certain neuronal types. These include NPY2R as a marker for all A-nociceptors [20] and CHRNA3 as a marker for MI neurons [22]. Notably, all of the above-mentioned studies are performed with cells from whole DRG tissue or used hindpaw skin as a model tissue and it is not evident whether the results reported can be extrapolated in the trigeminal system or in other tissues such as the muscle. Recently, Hockley *et al* reported single-cell sequencing of colonic sensory afferents and showed that the types of innervation varied significantly between L4, L5 and L6 DRG versus other lumbar or thoracic DRG neurons [49], emphasizing the importance of investigating the neuronal subtypes in different tissues and systems individually. Single-cell sequencing of whole TG neurons has been reported [50], where genes were classified into 13 clusters. However specific subgroups of TG neurons weren’t identified.

Due to dense sensory innervation brought in by the lingual nerve in the tongue and several disease conditions in the tongue that lead to a plethora of painful symptoms [1–6] [7, 8] [9–16](1–16), the current study determined the types of sensory innervation in the mouse tongue. Identification of sensory neuronal subtypes is crucial in a thorough understanding of the function and regulation of the lingual sensory neurons in normal as well as a diseased state as increased understanding of gene expression patterns will identify targets that may have therapeutic implications in treating lingual pain conditions. Additionally, since the tongue is a muscular tissue, data from the study will provide insight into types of innervation in other muscle tissues which to our knowledge has not been reported. We used the identified markers in the DRG tissue to classify and characterize the subtypes of sensory neurons carried by the lingual nerve to the tongue. We employed immunohistochemistry of transgenic reporter mouse lines expressing either the TDT or the GFP reporter under the promotor of each of the markers. Retrogradely labeled tongue neurons were evaluated for the frequency of colocalization with the reporter in each group as well as colocalization with either TRPV1, CGRP or Calbindin immunoreactivity. Our data demonstrated that the tongue is innervated with peptidergic and non-peptidergic neurons as measured by CGRP positivity (Fig 1A), that is in accordance to previously published studies [51]. In addition, major markers expressed in tongue neurons included TRPV1, 5HT3A, TrkC and Parvalbumin (Fig 1A). Expression of TRPV1 in the TG neurons innervating the tongue has also been reported previously [52, 53]. Calbindin was expressed in lingual neurons in a small proportion, suggesting that few rapidly adapting mechanically sensitive fibers may be present in the tongue (Fig 1A). In accordance with prior reports [21], significant proportion of 5HT3A neurons were peptidergic and also colocalized with TRPV1 (Fig 1B and 1E). Parvalbumin expressing neurons did not colocalize with TRPV1 or CGRP (Fig 1B and 1D) as reported previously [21, 23]. However, some calbindin-parv overlap was observed that has not been reported in the DRG. Known function of Parvalbumin (PV) is proprioception and proprioception via trigeminal afferents has been reported in various orofacial areas including tongue [54–56], jaw[57], eyelid [58], the face and vibrissae [59, 60]. While data about the source and structure of proprioceptors in the tongue are varied and may differ depending on species, reports suggest that trigeminal afferents may be partly involved in mediating tongue proprioception [54–57, 61]. As suggested previously, similar to the DRGs, PV expressing TG neurons may be expressed in proprioceptors or low-threshold mechanoreceptors [62]. However, it is noteworthy that differences lie between PV expressing DRG and TG neurons such as PV expressing TG neurons can innervate cells other than muscle spindles [60] and that not all PV positive TG neurons are TrkC positive, suggesting differential role of PV in TG neurons (Figs 1 and 4 and [60]. Accordingly, the precise role of PV expressing TG neurons innervating the tongue is yet to be determined.

Interestingly, unlike prior reports in the DRG neurons, a proportion of TrkC expressing neurons colocalized with CGRP, TRPV1 as well as Calbindin suggesting differences between TG and the DRG neurons (Fig 1B and 1C).

To confirm our anatomical results and further characterize lingual sensory neuronal subtypes, we performed single-cell PCR of the markers. We also tested for additional genes not tested with immunohistochemistry including CHRNA3, vGLUT3 and TRPA1. While vGLUT3 was not expressed in lingual neurons, CHRNA3 was observed in medium and small neurons potentially indicating the presence of MI neurons (Fig 2A and 2B). CHRNA3 has been reported to be expressed as an MI marker in C-neurons in the DRG and our data showed a similar expression pattern. An interesting feature of TG neurons that was observed, was that cell-size could not be used as a measure for identification of C, Aδ and Aβ neurons as not all small neurons were NFH negative and many medium and large neurons lacked NFH expression (Fig 1A). Therefore, CHRNA3, although expressed in medium neurons were unmyelinated C neurons without NFH expression. Of all the genes tested with single-cell PCR, the major markers expressed in lingual neurons were found to be similar (i.e 5HT3A, TrkC, parvalbumin and TRPV1) to that observed with immunohistochemistry. However, the number of cells expressing a few genes such as CGRP, NPY2R and TrkB were significantly higher with single-cell PCR than with immunohistochemistry. Several reasons can explain this discrepancy: 1) detection sensitivity of single-cell PCR is greater than immunohistochemistry; 2) potential translational control of the genes leading to differences in protein and RNA expression. It has been reported that same CALCA gene can undergo alternative splicing to form either calcitonin or CGRP [63]. It is also noteworthy that even though the number of CGRP expressing neurons in large, medium and small were similar, the intensity of gene expression was significantly higher in small neurons compared to medium and large neurons (Fig 3I); 3) differences in primer specificity of the gene; 4) presence of more than one isoform of a protein may limit detection of all isoforms with transgenic animals. It has been reported that TrkB can exists in its full-length form as well as a truncated form and could be controlled under separate promoters [64–68]. Another dissimilarity observed between single-cell results and immunohistochemistry was for the number of neurons expressing MRGPRA3. While we observed a small proportion of neurons expressing MRGPRA3 protein, no gene expression was observed in any cells tested. Similarly, frequency of MRGPRD expressing cells using immunohistochemistry was higher than that found with single-cell PCR. This could be due to potential differences in expression between males and females as gene expression was only evaluated in male mice whereas protein expression was tested for in males and female mice.

In further determining whether the major markers expressed in lingual sensory neurons belonged to the already identified subgroups, we analyzed the single-cell data for any overlapping markers as well as specific subgroups classified in the Usoskin et al and Patil et al study. Subgroups identified in tongue-innervating neurons included PEP2, NF3 and NF4/5 in large neurons, PEP1, PEP2, NF4 and NF5 in medium neurons and PEP1, PEP2 and NP3 in small neurons. Collectively these subgroups coincided with S2, N1, N3, M4, M5 and M7 types from Patil et al study. Similarities between TG neurons and DRG neurons were observed for TrkC, TRPV1, and parvalbumin. Our data showed that TRKC positive cells overlapped with CGRP, Parv and 5HT3A (Figs 2A, 4A and 4B). These genes are also shown to be expressed in Merkel Cells [30, 69, 70]. Whether the trigeminal afferents in the tongue express cells similar to Merkel cells is yet to be determined. So far, merkel cells in the tongue have been only reported in the taste buds[71, 72].

Many differences were found between lingual TG and DRG neurons such as 5HT3A was only identified to be in the PEP2 group in DRGs whereas our data showed that in addition to PEP2, it is also expressed in the NF3 group. While, the PEP2 expressing 5HT3A may play a role in sensitization of Ad-nociceptors as reported previously [73–77], NP3 expressing 5HT3A may contribute to tactile mechanoreception [78]. NPY2R was identified to be expressed primarily in the PEP2 and NP3 group when our data showed that it can be also expressed in a proportion of PEP1 group. It has been reported that NPY2R is a specific marker for Aβ- nociceptors in DRG neurons innervating mouse hindpaw [20]. However, Patil *et al* discussed that NPY2R population belonging to PEP2 group can be divided into 5HT3A positive and negative subgroups, of which 5HT3A+/NPY2R+ population may belong to Aδ-HTMR neurons whereas NPY2R+/5HT3A- subgroup may belong to Aβ-HTMR neurons [21]. Our data shows that both these neuronal types are expressed in the tongue (Fig 2A). Interestingly, function of NPY2R in PEP1 subtype is completely unknown. Expression of NPY2R in the NP3 subtype may suggest its role its role in inflammatory itch pathways. It would be interesting to explore the co-expression of NPY2R with other itch markers such as Nppb, IL31 and Cystlr2 [23]. Several conditions can produce itchy tongue and pruritus including cold sores, candidiasis, food allergies, stomatitis [79–81] and even in skin disorders such as Darier disease [82]. In the skin, itch responsive neurons belong to NP1-3 subgroups where NP1 and NP2 groups expressing MRGPRD and MRGPRA3 respectively, are believed to be exclusively expressed in the skin epidermis and may play a role in acute itch [23, 26, 27, 83]. Accordingly, we report that MRGRPD and MRGPRA3 is not expressed or expressed in very low proportions in lingual neurons. Therefore, itch in the tongue may be produced by the NP3 subgroup or may express other MRGPR genes not evaluated in this study. TrkB expression did not fall into any pre-identified groups and was seen to be expressed in large, medium and small neurons in significant numbers. Importantly, all the markers also fell into unidentified groups in addition to previously identified subgroups that are specific to lingual TG neurons. Single- cell sequencing of retrogradely labeled lingual neurons can provide further identification and characterization of sensory neuronal subgroups. Further, functional characterization and confirmation of markers and pathways of itch, proprioception and low-threshold mechanoreceptors in the tongue is warranted. Collectively, our data identifies sensory neuronal subtypes innervating the tongue that will allow to elucidate the physiological functions of these subtypes further providing insights into the mechanisms by which lingual sensory neurons are regulated in painful and itchy conditions.

## Supporting information

S1 Dataset Minimal data set for Figs 1, 2 and 3. (XLSX)
